# EGFR-Tyrosine Kinase Inhibitor Retreatment in Non-Small-Cell Lung Cancer Patients Previously Exposed to EGFR-TKI: A Systematic Review and Meta-Analysis

**DOI:** 10.3390/jpm14070752

**Published:** 2024-07-15

**Authors:** Isabella Michelon, Maysa Vilbert, Caio Ernesto do Rego Castro, Carlos Stecca, Maria Inez Dacoregio, Manglio Rizzo, Vladmir Cláudio Cordeiro de Lima, Ludimila Cavalcante

**Affiliations:** 1 Department of Medicine, Catholic University of Pelotas, Pelotas 96015-560, Brazil; 2 Massachusetts General Hospital Cancer Center, Division of Hematology/Oncology, Department of Medicine, Massachusetts General Hospital, Boston, MA 02114, USA; maysavilbert@gmail.com; 3 Department of Medicine, University of Brasilia, Brasília 70910-900, Brazil; 180117742@aluno.unb.br; 4 Department of Medicine, Parana Oncology Center, Curitiba 80030-200, Brazil; carlos.stecca@outlook.com; 5 Department of Medicine, University of Centro Oeste, Guarapuava 85040-167, Brazil; mdacoregio@unicentro.edu.br; 6 Cancer Immunobiology Laboratory, Instituto de Investigaciones en Medicina Traslacional, Universidad Austral-Consejo Nacional de Investigaciones Cientificas y Tecnologicas (CONICET), Buenos Aires 1428, Argentina; mrizzo@cas.austral.edu.ar; 7 Clinical Oncology Unit, Hospital Universitario Austral, Av. Presidente Perón 1500, (B1629ODT) Derqui-Pilar, Buenos Aires 1428, Argentina; 8 Department of Medical Oncology, AC Camargo Cancer Center, São Paulo 01509-010, Brazil; vladmir.lima@accamargo.org.br; 9 Department of Hematology and Medical Oncology, University of Virginia Comprehensive Cancer Center, Charlottesville, VA 22903, USA; mkj4gr@uvahealth.org

**Keywords:** non-small-cell lung cancer, NSCLC, epidermal growth factor receptor protein tyrosine kinase, EGFR, tyrosine kinase inhibitors, TKI, rechallenge, retreatment

## Abstract

We performed a systematic review and meta-analysis to assess the efficacy of EGFR-tyrosine kinase inhibitors (TKI) retreatment in advanced/metastatic non-small-cell lung cancer (NSCLC) patients. We systematically searched PubMed, Embase, Cochrane databases, ASCO, and ESMO websites for studies evaluating EGFR-TKI retreatment in advanced/metastatic NSCLC patients. All analyses were performed using R software (v.4.2.2). We included 19 studies (9 CTs and 10 retrospective cohorts) with a total of 886 patients. In a pooled analysis of all patients during retreatment with TKI, median OS was 11.7 months (95% confidence interval [CI] 10.2–13.4 months) and PFS was 3.2 months (95% CI 2.5–3.9 months). ORR was 15% (95% CI 10–21%) and DCR was 61% (95% CI 53–67%). The subanalysis by generation of TKI in the rechallenge period revealed a slightly better ORR for patients on 3rd generation TKI (*p* = 0.05). Some limitations include the high heterogeneity of some of the analyses and inability to perform certain subanalyses. Our results unequivocally support the benefit of EGFR-TKI rechallenge in EGFR-mutated NSCLC patients progressing on TKI treatment after a TKI-free interval. These findings may be especially valuable in areas where access to novel therapeutic drugs and clinical trials is limited.

## 1. Introduction

Non-small-cell lung cancer (NSCLC) accounts for roughly 85% of all lung cancer diagnoses [1]. About 10 to 30% of NSCLC patients harbor epidermal growth factor receptor (EGFR) mutations, most commonly exon 19 deletions and exon 21 point mutations [2]. EGFR mutations activate a signaling pathway essential to cellular growth and differentiation, as well as to tumor progression and carcinogenesis [3]. The identification of these genetic alterations makes EGFR an attractive therapeutic target [2]. Thus, in the past decade, EGFR inhibition with tyrosine kinase inhibitors (TKI)s remarkably changed the treatment paradigm of NSCLC patients with EGFR driver mutations [4,5]. The benefit of TKIs also extends to other tumors [6,7,8]. These agents have proven efficacy and are used in first-line settings in several cancers, such as chronic myeloid leukemia and advanced renal cell carcinoma. Moreover, a synergistic effect is noted when combining TKIs with other kinase inhibitors or immunotherapy, as seen in metastatic melanoma and human epidermal growth factor receptor 2 (HER2)-positive breast cancer.

EGFR-TKIs reversibly or irreversibly bind to the tyrosine kinase domain of EGFR, preventing downstream signaling and leading to cell cycle arrest and eventually cell death [9]. First-generation TKIs, namely erlotinib and gefitinib initially led the way in advanced EGFR-mutated NSCLC [10]. An early phase III study on stage III or IV NSCLC treated with erlotinib in the first-line setting reported a decrease of 84% in the rates of progression compared to chemotherapy (hazard ratio (HR) for disease progression or death, 0.16, 95% CI (confidence interval) 0.10–0.26; *p* < 0.0001) [4]. Afatinib, a second-generation TKI, was also superior to chemotherapy, with a median progression-free survival (PFS) of 11.1 versus 6.7 months (HR 0.49, 95% CI, 0.37–0.65; *p* = 0.001) [11].

The third-generation TKI, osimertinib, significantly improved progression-free survival of NSCLC T790M-positive patients compared to platinum-based chemotherapy in the phase III AURA trial (HR 0.30, 95% CI 0.23–0.41, *p* < 0.001) [12]. In the first-line FLAURA study, a phase III trial, osimertinib demonstrated greater efficacy over other EGFR-TKIs, placing it as the preferred TKI single agent for advanced NSCLC [13]. Median PFS was 18.9 months for the osimertinib arm versus 10.2 months for the comparator regimen [13]. Similarly, overall survival (OS) was 38.6 months versus 31.8 months at 3-year follow-up [14].

The combination of third-generation TKIs with other agents is also currently being studied [15]. The phase III FLAURA-2 trial reported an improved median PFS for the combination of osimertinib plus chemotherapy compared to osimertinib alone of 25.5 months versus 16.7 months, respectively (HR 0.62; 95% CI 0.49–0.79; *p* < 0.001) [15]. Another first-line phase III trial, MARIPOSA, investigated the combination of amivantamab (an EGFR-MET bispecific antibody) and Lazertinib (a third-generation EGFR-TKI) compared to osimertinib [16]. The combination achieved a longer median PFS of 23.7 months versus 16.6 months for osimertinib, with a trend towards better OS [16].

Despite this array of therapeutic options, most patients will inevitably experience resistance and disease progression on an EGFR-TKI [9]. Several studies of EGFR-TKIs approved for the typical EGFR-sensitive mutations support that a drug-free interval may restore TKI sensitivity [17,18,19,20,21,22]. Therefore, re-exposure to TKIs in patients who have previously progressed on such treatments may offer a new chance of tumoral response and better outcomes. However, these studies may have been limited by a small sample size. Moreover, there is a lack of evidence on the real-world effectiveness of this strategy. Thus, this systematic review and meta-analysis aimed to evaluate the efficacy of EGFR-TKI retreatment following a TKI-free interval in NSCLC patients who were initially treated with EGFR-TKIs in the first-line setting on a clinical trial and in the real-world setting.

## 2. Materials and Methods

This study was performed following the guidelines from the Cochrane Collaboration and the Preferred Reporting Items for Systematic Reviews and Meta-Analysis (PRISMA), and it was registered in the International Prospective Register of Systematic Reviews (PROSPERO) under the protocol number CRD42022383081 [23]. The complete PRISMA checklist can be found in Table S1.

### 2.1. Data Source and Search Strategy

A systematic search with no restriction regarding the publication date was performed in PubMed, Embase, and Cochrane databases in December of 2022. The search was last updated on 18 February 2024. The following combination of medical subject headings (MeSH) terms and boolean connectors were used: “non-small cell lung cancer” and (“TKI” or “tyrosine kinase inhibitors”) and (“retreatment” or “rechallenge”). The entire search strategy used as well as the version and link of each database can be found in Table S2. We have also searched references of all included studies and reviews.

### 2.2. Eligibility Criteria

All clinical trials and cohort studies analyzing the efficacy of EGFR-TKI retreatment in advanced/metastatic NSCLC patients who had progressed on a first-line TKI and on an interval-line of chemotherapy that reported any of the outcomes of interest were included. Studies analyzing TKI retreatment in the third or later line were included. Abstracts and conference presentations were also considered for inclusion. No restriction regarding the population size was made. Main exclusion criteria were as follows: (1) interruption of first-line TKI exclusively due to toxicity; (2) patients not treated with TKI in the first-line setting; (3) lack of interval chemotherapy between the first-line and rechallenge TKI; (4) lack of information regarding sequencing of TKI or chemotherapy; (5) outcomes of interest not reported; (6) case controls, case reports, case series, reviews, letters to the editor, and commentaries; and (7) manuscripts written in languages other than English.

### 2.3. Data Collection and Outcomes

Two authors (IM and CC) independently screened the articles using Zotero software and extracted the data from the studies included. A standardized Excel (version 16.86) sheet was used to collect each study’s population baseline characteristics and outcomes data. All inconsistencies between the authors were resolved by consensus or by consulting a third author (MV). We extracted data for pooled analysis on the following outcomes: (1) PFS; (2) OS; (3) objective response rate (ORR); (4) disease control rate (DCR); (5) duration of response (DOR); and (6) adverse events (AEs).

Subgroup analyses were performed according to (1) the design of the studies (CTs versus observational studies); (2) mutational status (*EGFR* sensitive mutations, T790M mutation, and mutational status unknown); (3) TKI choice in the rechallenge setting (same TKI versus a different TKI for the rechallenge); (4) first/second versus third-generation TKI; and (5) DCR in patients who initially achieved DCR with first TKI.

### 2.4. Quality Assessment

The ROBINS-I tool was used to assess the risk of bias in prospective clinical trials and observational cohort studies [24]. Two authors (IM and CC) independently completed the risk of bias assessment. Inconsistencies between the authors were resolved by consensus or by consulting a third author (MV). Following this protocol, each study was classified as low, moderate, serious, critical risk of bias or no information based on seven domains: bias due to confounding, selection of participants into the study, classification of interventions, deviations from intended intervention, missing data, in the measurement of outcomes and the selection of the reported result [24]. To verify the existence of publication bias, Egger’s test and funnel plots of individual study weights against point estimates were used.

### 2.5. Statistical Analysis

We performed proportional meta-analyses for binary outcomes and reported them in percentages of events with 95% CI. Logit-transformation of data was used when the individual study proportion was smaller than 0.2 or higher than 0.8. In the case of a study with zero events, we used the doubled-arcsine transformation. R software (version 4.2.2) was used to perform all statistical analyses. Pooled analysis of individual studies’ OS and PFS was carried out using medians with 95% CIs. The following packages were used: “metafor”; “meta”; “weight”; and “metagen”. I^2^ statistics were used to assess the heterogeneity; I^2^ > 25% were considered significant for heterogeneity. DerSimonian and Laird random-effects models were used in all analyses.

## 3. Results

### 3.1. Systematic Review

**Figure 1:**
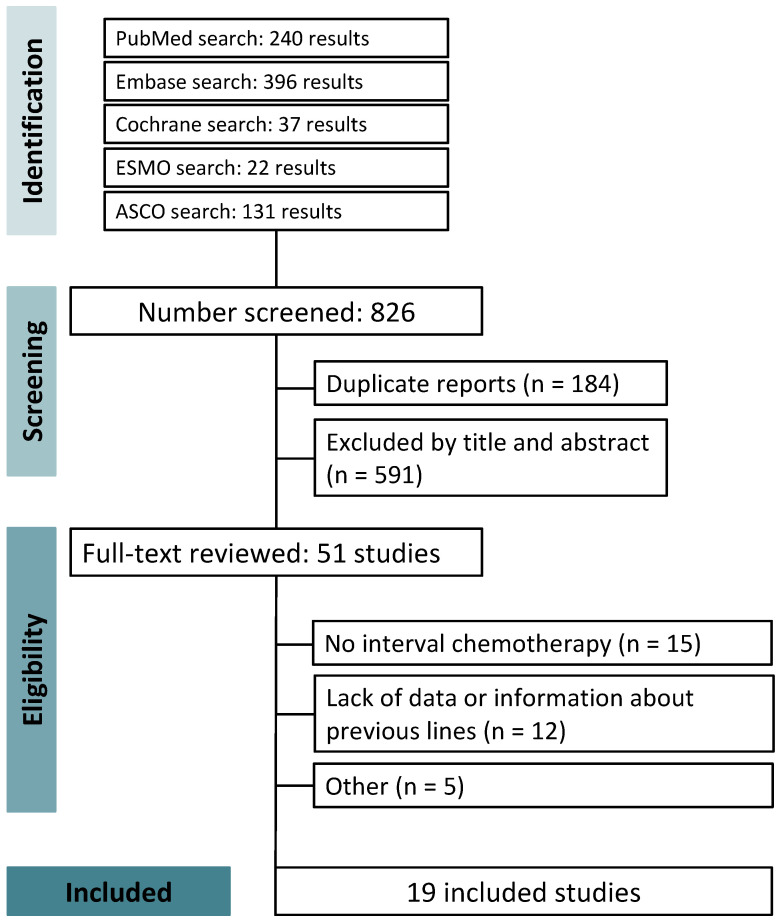
Preferred Reporting Items for Systematic Reviews and Meta-Analysis (PRISMA) flow diagram of study screening and selection. Blue vertical boxes indicate each stage of the screening, and the horizontal boxes present more detailed information about the process, including the steps performed in each stage. ASCO: American Society of Clinical Oncology; ESMO: European Society for Medical Oncology.

Initially, we identified 826 studies through our search strategy. After the removal of duplicates and exclusion by title and abstract, 51 studies were included for a comprehensive review. Finally, 19 studies met our eligibility criteria—nine prospective clinical trials and ten retrospective cohorts (Figure 1) [17,18,19,20,21,22,25,26,27,28,29,30,31,32,33,34,35,36,37]. A list of excluded studies after a comprehensive review can be found in the Supplementary Materials (Table S3) [38,39,40,41,42,43,44,45,46,47,48,49,50,51,52,53,54,55,56,57,58,59,60,61,62,63,64,65,66,67,68,69].

### 3.2. Baseline Characteristics

Of the 886 patients included, most were female (67.2%, n = 499/743), non-smokers (73.8%; n = 498/675), had adenocarcinoma (97.9%; n = 571/583), and received EGFR-TKI rechallenge as a third-line therapy (82.9%; n = 561/677). For most studies, data for metastatic sites and race or ethnicity were not available. The median age of each study ranged from 52 to 68 years. The median follow-up ranged from 7 to 38.9 months, and the TKI-free interval ranged from 5.9 to 13.8 months.

Osimertinib was used in the first-line and in the rechallenge setting in two studies. Gefitinib was the TKI of choice used in the first-line setting in seven studies. Of these, six studies also used gefitinib in the rechallenge. Erlotinib was used both in first-line and rechallenge in only one study. The ten remainder studies reported different TKIs in the first-line and in the rechallenge period. Baseline characteristics of included studies are presented in Table 1.

Most studies clearly defined that patients progressed on first-line TKI [17,18,19,20,21,25,26,28,29,30,31,32,33,34,36,37]. Among three studies, two did not specify the reason for TKI interruption, whereas one study stated that TKI rechallenge was administered after disease progression or unacceptable toxicity on first-line treatment [22,27,35].

### 3.3. Efficacy Outcomes

**Figure 2:**
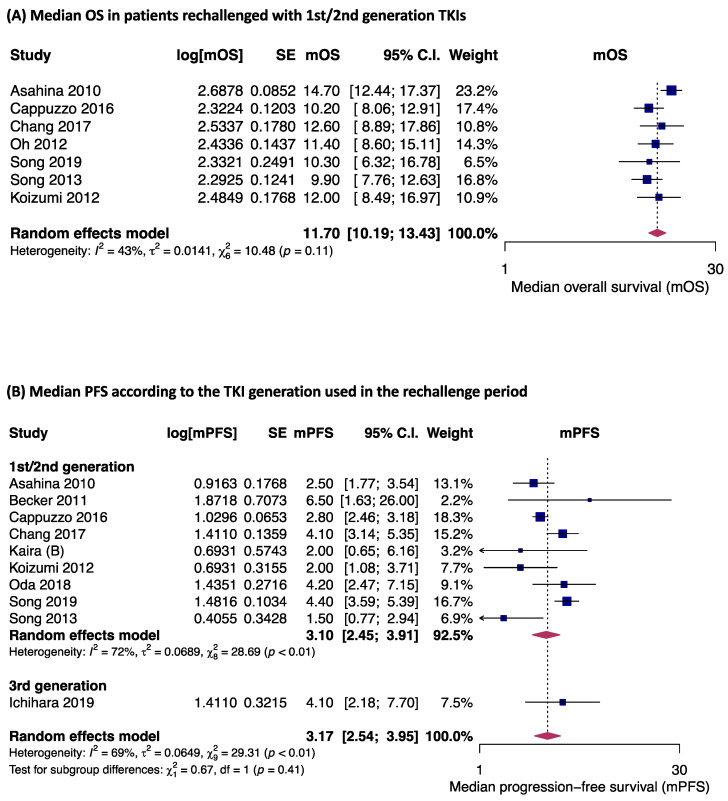
Median OS in patients rechallenged with first/second-generation TKI (**A**), and PFS according to the TKI generation used in the rechallenge period (**B**) [17,18,19,21,22,25,26,28,32,33,33]. Proportions for each trial are represented by a square, and the horizontal line crossing the squares indicates the 95% confidence interval. The diamonds represent the estimated overall effect of the meta-analysis based on random effects. CI: confidence interval; m: median; OS: overall survival; PFS: progression-free survival; TKI: tyrosine kinase inhibitor; SE: standard error. Kaira (B) refers to patients who did not receive PD-1 blockade before rechallenging [21].

In patients who were retreated with any TKI, the pooled analysis of seven studies revealed a median OS of 11.7 months (95% CI 10.2–13.4 months), and in the analysis of ten studies, the median PFS was 3.2 months (95% CI 2.5–3.9 months) (Figure 2). The subgroup analysis of nine studies considering only patients re-exposed with first- or second-generation TKIs revealed a PFS of 3.1 months (95% CI 2.4–3.9 months) (Figure 2B). Only one study analyzed patients retreated with 3rd generation TKIs and reported a PFS of 4.1 months (95% CI 2.2–7.7 months) (*p* = 0.41, Figure 2B). Survival data from studies that could not be pooled for survival analyses are presented in Table S4.

**Figure 3:**
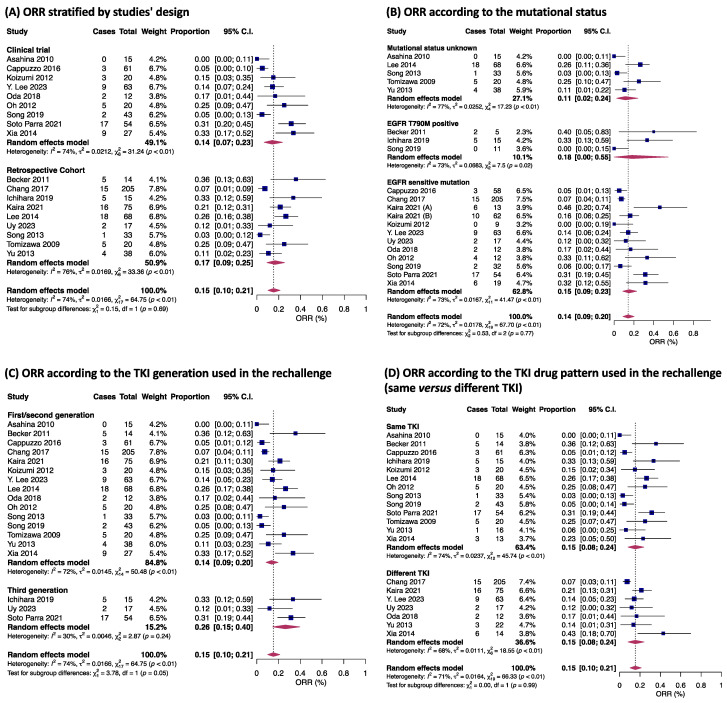
Objective response rate (ORR) according to the studies’ designs (**A**); ORR according to mutational status (**B**); ORR according to generation of TKI used in rechallenge (**C**); and ORR according to TKI drug pattern used in rechallenge (same vs. different TKI) (**D**) [17,18,19,20,21,22,25,26,28,29,30,31,32,33,34,35,36,37]. Proportions for each trial are represented by a square and the horizontal line crossing the squares indicates the 95% confidence interval. The diamonds represent the estimated overall effect of the meta-analysis based on random effects. CI: confidence interval; TKI: tyrosine kinase inhibitor. In Becker 2011, only one patient was treated with gefitinib and then erlotinib; all the others were first exposed to erlotinib both in the first-line treatment and rechallenge. Kaira (A): refers to patients treated with PD-1 blockade before TKI rechallenge; Kaira (B) refers to patients who did not receive PD-1 blockade before rechallenging [21].

Overall, the ORR across 18 studies was 15% (95% CI 10–21%), and no significant interaction was observed between observational studies and CTs (*p* = 0.69) (Figure 3A). In a subgroup analysis according to mutational profile, 556 patients harboring EGFR-sensitive mutations achieved an ORR of 15% (95% CI 9–23%), while 31 patients with the T790M mutation had an ORR of 18% (95% CI 0–55%). For the 174 patients with unknown mutational status, the ORR was 11% (95% CI 2–24%) (Figure 3B). No significant differences were observed between groups (*p* = 0.77) (Figure 3B). The analysis comparing rechallenge with different generations of TKI (15 studies on first/second- versus three studies on third-generation) revealed a borderline better ORR for 86 patients on third-generation (26% [95% CI 15–40%], I^2^ = 72%) TKI compared to 714 patients on first/second-generation (14% [95% CI 9–20%], I^2^ = 30%) (*p* = 0.05; Figure 3C). Individuals re-exposed to the same TKI as the first-line treatment presented with comparable responses to individuals treated with a different drug (*p* = 0.99) (Figure 3D).

**Figure 4:**
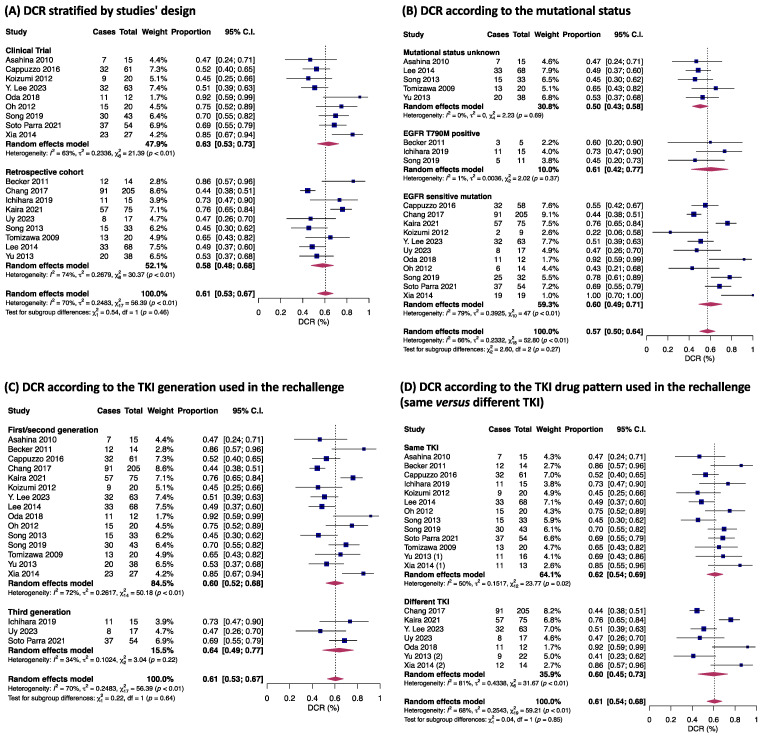
Disease control rate (DCR) according to studies´design (**A**); DCR according to mutational status (**B**); DCR according to generation of TKI used in rechallenge (**C**); and DCR according to TKI drug pattern used in rechallenge (same vs. different TKI) (**D**) [17,18,19,20,21,22,25,26,28,29,30,31,32,33,34,35,36,37]. Proportions for each trial are represented by a square and the horizontal line crossing the squares indicates the 95% confidence interval. The diamonds represent the estimated overall effect of the meta-analysis based on random effects. CI: confidence interval; TKI: tyrosine kinase inhibitors. In Becker 2011 only one patient was treated with gefitinib and then erlotinib, all the others were first exposed to erlotinib both in the first-line treatment and rechallenge. Kaira (A): refers to patients treated with PD-1 blockade before TKI rechallenge; Kaira (B) refers to patients who did not receive PD-1 blockade before rechallenging [21].

A pooled analysis of 18 studies showed that 61% of individuals (95% CI 53–67%) achieved a DCR, with no significant differences between observational studies and clinical trials (*p* = 0.46) (Figure 4A). In a subgroup analysis according to mutational status, 558 individuals harboring EGFR-sensitive mutations had a DCR of 60% (95% CI 49–71), whereas 31 with positive T790M had a DCR of 61% (95% CI 42–77%), and 174 with unknown mutational status, a DCR of 50% (95% CI 43–58%), with no significant differences seen among groups (*p* = 0.27) (Figure 4B). The analysis for DCR in 714 patients (16 studies) treated with first/second- versus 86 patients (three studies) on third-generation TKI showed a similar DCR of about 60% for both (*p* = 0.64; Figure 4C). The DCR was also similar for individuals treated with the same or a different TKI in the rechallenge period (*p* = 0.85; Figure 4D)

The subanalysis that included 284 patients who previously achieved DCR during first-line treatment revealed a DCR of 59% (95% CI 49–68%) with rechallenge (Figure S1). The median duration of response to TKI rechallenge was described in only five studies, and it ranged from 2.2 to 4.4 months, as presented in Table S5.

Analyses stratified by TKI-free intervals were reported in five studies, and the information available is described in Table S6. In total, 3 studies with 52 patients found no correlation between TKI-free intervals and response or survival rates [19,28,34]. In 2 studies, 273 patients rechallenged after a longer TKI-free period had better response or survival rates compared to shorter intervals [22,30].

### 3.4. Adverse Events

The most common any-grade adverse events were skin toxicity and diarrhea (Figure S2). For both, the incidence of grade 1–2 AEs was significantly more common than grade 3 or higher (*p* ≤ 0.01) (Figure S2).

### 3.5. Quality Assessment

Four studies were judged to be at serious risk of bias, mostly due to the retrospective design and lack of adjustment for confounding factors (Table S7) [21,26,31,34]. The other 15 studies (6 retrospective and 9 prospective non-randomized trials) were at moderate risk of bias [17,18,19,20,22,25,27,28,29,30,32,33,35,36,37]. In the funnel plot analysis for ORR, a wide distribution of studies was seen (Figure S4). However, the Egger’s test showed no indication of publication bias (z = 0.93; *p* = 0.35).

## 4. Discussion

To the best of our knowledge, this systematic review and meta-analysis is the first to evaluate advanced NSCLC patients undergoing TKI retreatment after progression on initial TKI and interval line of chemotherapy. Our comprehensive meta-analysis revealed a median overall survival of about a year for retreatment in the third line or later, an ORR of 15%, and a favorable DCR of 61%. Patients who previously achieved DCR in their initial TKI therapy exhibited a noteworthy 59% DCR upon retreatment. Additionally, those retreated with third-generation TKI demonstrated the highest ORR at 26% and a DCR of 64%. Among patients harboring EGFR-sensitive mutations, 60% DCR was observed with TKI rechallenge. Retreatment with either the same or a different TKI demonstrated comparable efficacy, with no significant differences observed between study designs, mutational statuses, or whether patients were re-exposed to the same first-line TKI or a different one. The safety profile was similar to that observed in previous studies on TKI monotherapy in earlier settings [4,11,12].

In NSCLC patients harboring EGFR mutations, a significant benefit over chemotherapy was observed with EGFR-TKI in the first-line setting [4,11,70,71,72]. The most remarkable predictor of TKI response is EGFR mutational status, particularly exon 19 deletion and L858R point mutation in exon 21 [73,74,75]. A phase III trial reported a 70% improvement in PFS in patients receiving gefitinib compared to those on carboplatin-paclitaxel [70]. The EGFR-TKI group also exhibited higher response rates (89.5% versus 79.8%). Despite initial positive responses, most patients eventually face disease progression within the first or second year of treatment due to acquired resistance [76].

The clinical benefit of EGFR-TKIs in advanced NSCLC patients is influenced by genetic, clinical, and pathological factors [73,77]. Compared to other histological types, adenocarcinoma frequently presents with a better prognosis [73,74,75]. Non-smokers, females, and patients of Asian descent are known to derive the highest benefit from EGFR-TKIs [78,79]. Moreover, previous lines of therapy and resistance mechanisms (e.g., acquired T790M mutation) may influence the effectiveness of subsequent strategies [80,81]. In this meta-analysis, a high number of patients had adenocarcinoma, were female, and were non-smokers. However, we could not perform subgroup analyses based on all these factors due to lack of patient level data from individual studies.

In EGFR-mutated NSCLC, alterations within the EGFR domains and downstream pathways drive malignant cell survival. Emerging evidence indicates that NSCLC may harbor mutated and non-mutated EGFR clones in the same tumor tissue [82]. During TKI treatment, sensitive cells are suppressed, and eventually, resistant cells begin to proliferate, which are then targeted by subsequent therapies [17,83]. Following a period free of TKIs, EGFR-sensitive clones are prone to re-growth [36]. As tumors regain sensitivity, TKI re-treatment may once again effectively target tumoral cells [17,18,36]. Another rationale for TKI rechallenge benefit relies on oncogene addiction [84]. This theory explains that despite the extensive genetic and epigenetic modifications seen in several tumors, blocking just one mutation can inhibit cancer cells from growth and proliferation [84]. This is attributed to the fact that tumor survival is often linked to one or few driver oncogenes, and targeting them may more precisely prevent disease progression [84].

Drug rechallenge, particularly with targeted therapies, has arisen as a potential strategy to increase disease control in refractory patients who have progressed on multiple lines of therapy. Reintroduction of the same therapy has been reported in melanoma, colorectal cancer, and renal cell carcinoma [85,86,87,88,89,90]. A meta-analysis including 400 patients with BRAF-mutated advanced melanoma showed a 65% DCR with rechallenge of BRAF and MEK inhibitors in later lines [91]. It also showed a median survival of 9.8 months and that a targeted therapy free interval of 6 months or more was associated with a greater DCR [91]. The largest cohort included in our current meta-analysis explored EGFR-TKI rechallenge in 205 advanced or metastatic NSCLC patients [22]. Median OS and PFS were 12.6 and 4.1 months, respectively, and the DCR was 44.4%. Overall, our pooled analyses found a median OS and PFS of 11.7 and 3.2 months, respectively, and a 61% DCR. Additionally, the AEs most often reported among studies were skin toxicity and diarrhea, consistent with prior studies on EGR-TKI in the first line [4,11,12]. For both, grade 1–2 events were significantly more common than grade 3. Thus, our findings support retreatment with EGFR-TKI as a viable therapeutic strategy for patients who progressed on previous TKI and other lines of therapy. Importantly, the rechallenge appears to be well tolerated, without additional toxicity. This is especially important for low- and middle-income countries with more limited options, where the newest therapies and clinical trials may not be readily accessible.

Multiple mechanisms are involved with tumoral-acquired resistance. However, most are not well understood [76,92]. In the scenario of EGFR-mutated NSCLC, acquired resistance can be classified as target or target-dependent and off-target or target-independent [93,94,95]. The first develops within the kinase domain of EGFR. The latter involves targets other than the kinase domain (e.g., bypass mechanisms and phenotypic transformation) [93,94,95]. Off-target resistance is more frequently seen in patients on third-generation TKI, whereas on-target resistance is seen in patients on first- and second-generation TKI [93,94,95]. In contrast, some evidence has shown that over 50% of patients with acquired resistance to first or second-generation TKIs develop new mutations within the drug domain [76].

One of the most common resistance on-target mutations to first- and second-generation TKIs is T790M, which affects the EGFR binding and enzymatic site [76,82]. Previous studies have consistently associated the presence of T790M with a poor prognosis [5,26]. However, findings from the AURA3 trial demonstrated that NSCLC patients harboring T790M mutations and treated with osimertinib achieved a great response, with an ORR of 71% after progressing on first-line EGFR-TKI therapy [12]. This underscores the sensitivity of T790M to the 3rd generation TKI osimertinib. In our study, the subgroup analysis of 31 patients carrying T790M mutations previously treated with various TKIs revealed a DCR of 61%.

Osimertinib has consistently demonstrated notable efficacy in the rechallenge scenario [20,31,35]. In our search, we found three studies assessing the response of osimertinib in this specific context, encompassing two retrospective studies and one prospective trial [20,31,35]. Uy et al. and Ichihara et al., both observational retrospective cohorts, reported response rates ranging from 12 to 33% and DCR spanning from 47 to 74%, whereas OSIRS, a phase II study, reported ORR and DCR values of 31.5% and 69%, respectively [20,31,35]. Our study found an ORR of 26% and a DCR of 64% in the subgroup of patients rechallenged with osimertinib.

One study included patients who were retreated with afatinib [19]. In the phase II trial conducted by the Okayama Lung Cancer Study Group, 12 patients retreated with afatinib achieved an ORR of 17% and a remarkable DCR of 92% [19]. Results from an ongoing phase II multicenter study—the REAL study, with a target enrollment of 30 patients—are awaited to better address the role of afatinib rechallenge in patients progressing after first-line osimertinib (UMIN000049225) [67].

Some ways to overcome resistance include combinations of TKI with other targeted therapies, chemotherapy, and local therapies [5,9,76,96,97]. Emerging methods, such as circulating tumor DNA/RNA and sequencing of oncogenic drivers, may identify targets early in the scenario of acquired resistance and provide some directions for better treatment selection [83,98]. Results from the ongoing ORCHARD, a phase II study investigating strategies based on the tumor molecular profile for advanced NSCLC patients who progressed on first-line osimertinib, may address some of these questions (NCT03944772) [99].

Despite recent advances, treating metastatic EGFR mutant NSCLC patients progressing on first-line TKI is still challenging [5]. The MARIPOSA-2 trial has investigated a promising combination therapy of amivantamab-chemotherapy plus lazertinib or amivantamab-chemotherapy compared to chemotherapy alone in patients after progression to osimertinib and has shown improved PFS and intracranial PFS [100]. Another very important question is regarding the continuation of osimertinib following first-line progression [101,102]. This has been investigated in ongoing clinical trials combining osimertinib with several agents [103]. Regarding third-line therapy for EGFR-mutant NSCLC patients, previously treated with EGFR-TKI and platinum-based chemotherapy, a novel antibody-drug conjugate patritumab deruxtecan, or HER3-DXd, has been evaluated in the phase II HERTHENA-Lung01 [104]. Patients on HER3-DXd achieved an ORR of 29.3%, a median PFS of 5.5 months, and a median OS of 11.9 months [104]. Our study of TKI rechallenge in the third or later line showed a similar median OS (11.7 months) to HERTHENA-Lung01, showing that TKI retreatment may benefit patients with limited therapeutic options after disease progression.

The therapeutic landscape of EGFR-mutated NSCLC has dramatically changed with the addition of promising new therapies and advances in precision medicine. Nonetheless, these groundbreaking discoveries are accompanied by vast disparities between patients in different countries or from different socio-economic backgrounds [105,106,107,108,109]. Middle- and low-income countries have limited access to targeted therapies, significant barriers in performing molecular diagnostic tests, and worse cancer treatment outcomes compared to high-income countries [105,106,107,108,109]. Additionally, pre-existing discrepancies in cancer care are likely to widen as treatment paradigms grow increasingly more complex and costly [109]. Our meta-analysis demonstrating the benefit of EGFR-TKI in the rechallenge scenario may be of special value in locations with limited access to new therapeutic developments, but research and initiatives to increase access to cancer therapies and oncological care around the world should grow concurrently with new drug development efforts.

While our study provides valuable insights, it is essential to acknowledge certain limitations. The unavailability of data from individual studies hindered our ability to perform subanalyses based on crucial factors such as drug-free time or the number of chemotherapy cycles. Moreover, appreciable heterogeneity was observed in some analyses, likely related to inclusion of observational and small-size studies. Nonetheless, in recognition of these limitations, we implemented random-effects models across all analyses to mitigate potential biases. Furthermore, we conducted separate analyses based on the studies’ design and other relevant subgroups to enhance our findings’ robustness and reliability. Further prospective studies on EGFR-TKI rechallenge should focus on better stratification of patients and assessment of response according to important clinicopathological factors (e.g., age, gender, ethnicity, smoking, mutational status, previous lines of therapies, and TKI-free interval).

## 5. Conclusions

Our meta-analysis represents the first comprehensive assessment of TKI re-exposure in patients with NSCLC who progressed on both first-line TKI and subsequent therapy. The results suggest that advanced EGFR-mutated NSCLC patients who experience treatment failure with initial TKI treatment may derive benefit from rechallenge with an EGFR-TKI following a TKI-free interval. Importantly, our analysis indicates that the effectiveness of re-exposure remains consistent whether with the same or a different TKI. These findings contribute valuable insights into potential therapeutic strategies for this patient population, especially when access to novel therapeutic drugs and clinical trials is limited.

## Figures and Tables

**Table 1:** Design and characteristics of studies included in the meta-analysis.

Study	Design	Location	N	Female N (%)	Median Age in Years (Range)	Non-Smokers N (%)	ECOG PS N (%)		Initial TKI	Rechallenge TKI	Rechallenge Line of Treatment N (%)		TKI-Free Interval in Months ^a^	Median Follow-Up Interval in Months (Range)
							0–1	≥2			3rd	≥4th		
Asahina 2010 [25]	Phase II CT	Japan	16	13 (81)	66.5 (53–79)	11 (69)	14 (87.5)	2 (12.5)	Gef	Gef	2 (12.5)	14 (87.5)	≤6 mos: 8 (50) 6–12 mos: 6 (38) ≥12 mos: 2 (13) ^b^	14.7
Becker 2011 [26]	Retrospective cohort	Netherlands	14	9 (64)	55 (39–70)	NA	NA	NA	Erl ^c^	Erl ^c^	14 (100)	0	9.5 (3–36)	9 (1.5–16+)
Cappuzzo 2016 [12]	Phase II CT	Italy	61	45 (73.8)	67 (40–86)	41 (67.2) ^d^	50 (82)	10 (8) ^e^	Gef	Gef	61 (100)	0	NA	9 (0–19)
Chang 2017 [22]	Retrospective cohort	Taiwan	205	129 (62.9)	61.8 (31.4–92.9)	150 (73.2) ^d^	34	171	Gef, Erl, Afa	Gef, Erl, Afa	205 (100)	0	NA	NA
Chen 2016 [27]	Retrospective cohort	Taiwan	80	NA	NA	NA	NA	NA	NA	NA	NA	NA	<3 mos: 16 (20) 3–6 mos: 25 (31.3) >6 mos: 39 (48.8) ^b^	7 (NA)
Ichihara 2019 [20]	Retrospective cohort	Japan	17	11 (65.7)	68 (43–78)	17 (100)	11	6	Osi	Osi	NA	NA ^f^	5.9 (0.6–19)	NA
Kaira 2021 [21]	Retrospective cohort	Japan	75	46 (61.3)	65 (39–79)	43 (57.3)	56 (74.7)	19 (25.3)	Gef, Erl, Afa, Osi	Gef, Erl, Afa	13 (17.3)	62 (82.7)	NA	NA
Koizumi 2012 [28]	Phase II CT	Japan	20	17 (85)	61 (41–81)	18 (90) ^d^	14 (70)	6 (30)	Gef	Gef	3 (15)	17 (85)	NA	NA
Y. Lee 2023 [29] *	Phase II CT	South Korea	63	NA	65 (NA)	NA	NA	NA	Gef, Erl	Gef, Erl	NA	NA	NA	NA
Lee 2014 [30]	Retrospective cohort	South Korea	68	54 (79.4)	NA	57 (83.8) ^d^	34 (50)	34 (50)	Gef, Erl	Gef, Erl	68 (100)	0	9.8 (2.1–22.4)	38.9 (34.7–44.5)
Oda 2018 [19]	Phase II CT	Japan	12	9 (75)	67.5 (52–86)	1 (8.3) ^d^	12 (100)	0	Gef, Erl, Afa	Afa	NA	NA ^g^	9.6 (2.5–37.8)	10.2 (4.3–31.9)
Oh 2012 [32]	Phase II CT	South Korea	23	20 (86.9)	65 (45–79)	21 (91.3) ^d^	17 (73.9)	6 (26)	Gef	Gef	0	23 (100)	7 (2.5–25)	8.6 (0.4–20.8)
Song 2019 [17]	Phase II CT	China	43	30 (69.8)	57 (46–77)	36 (83.7)	37 (86)	6 (14)	Gef	Gef	43 (100)	0	NA	NA
Song 2013 [33]	Retrospective cohort	China	33	17 (51.5)	57.9 (32–76)	23 (69.7)	22 (66.6)	11 (33.4)	Erl, Gef	Erl, Gef	33 (100)	0	NA	30.2 (6.7–56)
Soto Parra 2021 [35] *	Phase II CT	Italy	54	37 (68.5)	NA	NA	54 (100)	0	Osi	Osi	54 (100)	0	NA	NA
Tomizawa 2009 [34]	Retrospective cohort	Japan	20	17 (85)	67 (34–79)	15 (75)	18 (90)	2 (10)	Gef	Gef	NA	NA	7.2 (1.4–27.3)	NA
Uy 2023 [31] *	Retrospective cohort	United States	17	11 (65)	62 (42–73)	13 (77)	12 (70.6)	5 (29.4)	Erl, Afa, Osi	Osi	NA	NA ^h^	13.8 (3.2–50.7)	NA
Xia 2014 [36]	Phase II CT	China	27	12 (44.4)	NA	24 (88.9) ^d^	20 (74)	7 (26)	Gef, Erl, Ico	Gef, Erl, Ico	27 (100)	0	NA	NA
Yu 2013 [37]	Retrospective cohort	China	38	22 (57.9)	Gef-group:54 (50.6–57.4) Erl-group: 52 (42.2–61.8)	28 (73.7)	NA	NA	Gef	Gef, Erl	38 (100)	0	Gef-group:4 (NA) Erl-group: 5 (NA)	NA

Afa: afatinib; CT: clinical trial; ECOG PS: Eastern Cooperative Oncology Group Performance Status; Erl: erlotinib; Gef: gefitinib; Ico: icotinib; NA: not available; mos: months; N: number of patients; TKI: tyrosine kinase inhibitors; USA: United States of America; Osi: osimertinib; * abstracts from conferences. ^a^: TKI-free interval is presented as median (range) unless indicated otherwise; ^b^: in these studies, TKI-free interval was presented in three categories, followed by the number of patients in each category, and percentages are presented between parenthesis; ^c^: in the study Becker 2011, most patients (13 out of 14) received erl in the initial and rechallenge treatment, alone or in combination with other agents, and only one patient received gef as the initial treatment and was rechallenged with erlotinib; ^d^: never smokers; ^e^: information about ECOG PS not available for one patient; ^f^: in this study, the median number of previous therapies was five; ^g^: in this study, the median number of therapies was four; ^h^: in this study the median number of therapies was three.

## Data Availability

All research data presented in this study is accessible to the corresponding author upon request.

## Supplementary Materials

The following supporting information can be downloaded at: https://www.mdpi.com/article/10.3390/jpm14070752/s1; Table S1—Preferred Reporting Items for Systematic Reviews and Meta-Analysis (PRISMA) checklist for the manuscript (A), and the abstract (B); Table S2—Full search strategy used in each database: (A) Pubmed; (B) Cochrane; (C) Embase; (D) ESMO; (E) ASCO; Table S3—List of studies excluded after full review; Table S4—Median OS (A) and median PFS (B) of individual studies for NSCLC patients treated with EGFR-rechallenge; Table S5—Median duration of response of TKI rechallenge according to individual studies; Table S6—Efficacy outcomes according to the TKI-free interval between the first-line and rechallenge TKI for individual studies; Table S7—Quality assessment of studies using the risk of bias summary for non-randomized studies (ROBINS-I) tool. Figure S1—DCR during rechallenge in the subgroup of patients who previously achieved DCR with initial TKI; Figure S2—Frequency of adverse events stratified by grade: (A) skin toxicity; and (B) diarrhea; and Figure S3—Funnel plot analysis of objective response rate (ORR) in NSCLC patients rechallenged with TKI.

## Abbreviations

AEs, adverse events; Afa, afatinib; ASCO, American Society of Clinical Oncology; CI, confidence interval; CTs, clinical trials; DCR, disease control rate; DOR, duration of response; ECOG PS, Eastern Cooperative Oncology Group Performance Status; EGFR, epidermal growth factor receptor; Erl, erlotinib; ESMO, European Society for Medical Oncology; Gef, gefitinib; HR, hazard ratio; HER2, human epidermal growth factor receptor 2; Ico, icotinib; ICI, immune checkpoint inhibitors; MeSH, medical subject headings; mos, months; NSCLC, non-small cell lung cancer; NA, not available; N, number of patients; ORR, objective response rate; Osi, osimertinib; OS, overall survival; PRISMA, preferred reporting items for systematic reviews and meta-analyses.
