# Supplementary material for: EGFR-Tyrosine Kinase Inhibitor Retreatment in Non-Small-Cell Lung Cancer Patients Previously Exposed to EGFR-TKI: A Systematic Review and Meta-Analysis
Source: J Pers Med. 2024 Jul 15;14(7):752. doi: 10.3390/jpm14070752 (PMC11277985; doi:10.3390/jpm14070752)
Supplement: Supplementary file 1 [file jpm-14-00752-s001.zip › jpm-3075131-supplementary.pdf]

EGFR-tyrosine kinase inhibitor retreatment in non-small cell lung cancer patients previously exposed to EGFR-TKI: a systematic review and meta-analysis

Supplementary material

Table of Contents

Table S1 Preferred Reporting Items for Systematic Reviews and Meta-Analysis (PRISMA) Checklist for the Manuscript (A), and for the Abstract (B). **.......... 1**

(A) Manuscript Prisma Checklist 1

(B) Abstract Prisma Checklist 4

Table S2 Full search strategy used in each database: (A) Pubmed; (B) Cochrane; (C) Embase; (D) ESMO; (E) ASCO **.......... 5**

(A) Pubmed 5

(C) Embase 5

(D) ESMO 5

(E) ASCO 5

Table S3 List of studies excluded after full review **.......... 6**

Table S4 Median OS (A) and median PFS (B) of individual studies for NSCLC patients treated with EGFR-rechallenge **.......... 8**

(A) Median OS 8

(B) Median PFS 9

Table S5 Median duration of response of TKI rechallenge according to individual studies **.......... 10**

Table S6 Efficacy outcomes according to the TKI-free interval between the first-line and rechallenge TKI for individual studies **.......... 10**

Table S7 Quality assessment of studies using the risk of bias summary for non-randomized studies (ROBINS-I) tool **.......... 11**

Figure S1 DCR during rechallenge in the subgroup of patients who previously achieved DCR with initial TKI **.......... 12**

Figure S2 Frequency of adverse events stratified by grade: (A) skin toxicity; and (B) diarrhea **13**

Figure S3 Funnel plot analysis of objective response rate (ORR) in NSCLC patients rechallenged with TKI **14**

Tables

Table S1 Preferred Reporting Items for Systematic Reviews and Meta-Analysis (PRISMA) Checklist for the Manuscript (A), and for the Abstract (B).

(A) Manuscript Prisma Checklist

Section and Topic	Item #	Checklist item	Location where item is reported
**TITLE**			
**Title**	**1**	Identify the report as a systematic review.	**Page 1**
**ABSTRACT**			
**Abstract**	**2**	See the PRISMA 2020 for Abstracts checklist.	**Table S1B**
**INTRODUCTION**			
**Rationale**	**3**	Describe the rationale for the review in the context of existing knowledge.	**Pages 1, 2**
**Objectives**	**4**	Provide an explicit statement of the objective(s) or question(s) the review addresses.	**Pages 1, 2**
**METHODS**			
**Eligibility criteria**	**5**	Specify the inclusion and exclusion criteria for the review and how studies were grouped for the syntheses.	**Page 3**
**Information sources**	**6**	Specify all databases, registers, websites, organizations, reference lists and other sources searched or consulted to identify studies. Specify the date when each source was last searched or consulted.	**Pages 2, 3**
**Search strategy**	**7**	Present the full search strategies for all databases, registers and websites, including any filters and limits used.	**Table S2**
**Selection process**	**8**	Specify the methods used to decide whether a study met the inclusion criteria of the review, including how many reviewers screened each record and each report retrieved, whether they worked independently, and if applicable, details of automation tools used in the process.	**Page 3**
**Data collection process**	**9**	Specify the methods used to collect data from reports, including how many reviewers collected data from each report, whether they worked independently, any processes for obtaining or confirming data from study investigators, and if applicable, details of automation tools used in the process.	**Page 3**
**Data items**	**10a**	List and define all outcomes for which data were sought. Specify whether all results that were compatible with each outcome domain in each study were sought (e.g. for all measures, time points, analyses), and if not, the methods used to decide which results to collect.	**Page 3**
	**10b**	List and define all other variables for which data were sought (e.g. participant and intervention characteristics, funding sources). Describe any assumptions made about any missing or unclear information.	**Page 3**
**Study risk of bias assessment**	**11**	Specify the methods used to assess risk of bias in the included studies, including details of the tool(s) used, how many reviewers assessed each study and whether they worked independently, and if applicable, details of automation tools used in the process.	**Page 3**

**Effect measures**	**12**	Specify for each outcome the effect measure(s) (e.g. risk ratio, mean difference) used in the synthesis or presentation of results.	**Pages 3, 4**
**Synthesis methods**	**13a**	Describe the processes used to decide which studies were eligible for each synthesis (e.g. tabulating the study intervention characteristics and comparing against the planned groups for each synthesis (item #5)).	**NA**
	**13b**	Describe any methods required to prepare the data for presentation or synthesis, such as handling of missing summary statistics, or data conversions.	**NA**
	**13c**	Describe any methods used to tabulate or visually display results of individual studies and syntheses.	**Page 3**
	**13d**	Describe any methods used to synthesize results and provide a rationale for the choice(s). If meta-analysis was performed, describe the model(s), method(s) to identify the presence and extent of statistical heterogeneity, and software package(s) used.	**Page 4**
	**13e**	Describe any methods used to explore possible causes of heterogeneity among study results (e.g. subgroup analysis, meta-regression).	**Page 3**
	**13f**	Describe any sensitivity analyses conducted to assess robustness of the synthesized results.	**NA**
**Reporting bias assessment**	**14**	Describe any methods used to assess risk of bias due to missing results in a synthesis (arising from reporting biases).	**NA**
**Certainty assessment**	**15**	Describe any methods used to assess certainty (or confidence) in the body of evidence for an outcome.	**NA**
RESULTS			
**Study selection**	**16a**	Describe the results of the search and selection process, from the number of records identified in the search to the number of studies included in the review, ideally using a flow diagram.	**Figure 1**
	**16b**	Cite studies that might appear to meet the inclusion criteria, but which were excluded, and explain why they were excluded.	**Table S3**
**Study characteristics**	**17**	Cite each included study and present its characteristics.	**Table 1**
**Risk of bias in studies**	**18**	Present assessments of risk of bias for each included study.	**Table S7**
**Results of individual studies**	**19**	For all outcomes, present, for each study: (a) summary statistics for each group (where appropriate) and (b) an effect estimate and its precision (e.g. confidence/credible interval), ideally using structured tables or plots.	**Pages 4-12**
**Results of syntheses**	**20a**	For each synthesis, briefly summarise the characteristics and risk of bias among contributing studies.	**Table S7**

	**20b**	Present results of all statistical syntheses conducted. If meta-analysis was done, present for each the summary estimate and its precision (e.g. confidence/credible interval) and measures of statistical heterogeneity. If comparing groups, describe the direction of the effect.	**Pages 8-12**
	**20c**	Present results of all investigations of possible causes of heterogeneity among study results.	**Page 8-12**
	**20d**	Present results of all sensitivity analyses conducted to assess the robustness of the synthesized results.	**NA**
**Reporting biases**	**21**	Present assessments of risk of bias due to missing results (arising from reporting biases) for each synthesis assessed.	**NA**
**Certainty of evidence**	**22**	Present assessments of certainty (or confidence) in the body of evidence for each outcome assessed.	**NA**
DISCUSSION			
**Discussion**	**23a**	Provide a general interpretation of the results in the context of other evidence.	**Page**
	**23b**	Discuss any limitations of the evidence included in the review.	**Page 12**
	**23c**	Discuss any limitations of the review processes used.	**Page 14**
	**23d**	Discuss implications of the results for practice, policy, and future research.	**Page 14**
OTHER INFORMATION			
**Registration and protocol**	**24a**	Provide registration information for the review, including register name and registration number, or state that the review was not registered.	**Page 3**
	**24b**	Indicate where the review protocol can be accessed, or state that a protocol was not prepared.	**Page 3**
	**24c**	Describe and explain any amendments to information provided at registration or in the protocol.	**NA**
**Support**	**25**	Describe sources of financial or non-financial support for the review, and the role of the funders or sponsors in the review.	**Pages 14**
**Competing interests**	**26**	Declare any competing interests of review authors.	**Pages 15**
**Availability of data, code and other materials**	**27**	Report which of the following are publicly available and where they can be found: template data collection forms; data extracted from included studies; data used for all analyses; analytic code; any other materials used in the review.	**Page 14**

NA: not available

(B) Abstract Prisma Checklist

Section and Topic	Item #	Checklist item	Reported (Yes/No)
**TITLE**			
**Title**	**1**	Identify the report as a systematic review.	**Yes**
BACKGROUND			
**Objectives**	**2**	Provide an explicit statement of the main objective(s) or question(s) the review addresses.	**Yes**
METHODS			
**Eligibility criteria**	**3**	Specify the inclusion and exclusion criteria for the review.	**Yes**
**Information sources**	**4**	Specify the information sources (e.g. databases, registers) used to identify studies and the date when each was last searched.	**Yes**
**Risk of bias**	**5**	Specify the methods used to assess risk of bias in the included studies.	**No**
**Synthesis of results**	**6**	Specify the methods used to present and synthesise results.	**Yes**
RESULTS			
**Included studies**	**7**	Give the total number of included studies and participants and summarise relevant characteristics of studies.	**Yes**
**Synthesis of results**	**8**	Present results for main outcomes, preferably indicating the number of included studies and participants for each. If meta-analysis was done, report the summary estimate and confidence/credible interval. If comparing groups, indicate the direction of the effect (i.e. which group is favoured).	**Yes**
DISCUSSION			
**Limitations of evidence**	**9**	Provide a brief summary of the limitations of the evidence included in the review (e.g. study risk of bias, inconsistency and imprecision).	**No**
**Interpretation**	**10**	Provide a general interpretation of the results and important implications.	**Yes**
OTHER			
**Funding**	**11**	Specify the primary source of funding for the review.	**No**
**Registration**	**12**	Provide the register name and registration number.	**No**

Table S2 Full search strategy used in each database: (A) Pubmed; (B) Cochrane; (C) Embase; (D) ESMO; (E) ASCO

(A) Pubmed
("NSCLC" or "Non-small cell lung cancer" or "non-small cell lung cancer") and ("TKI" or "tyrosine kinase inhibitors" or "anti-EGFR" or "EGFR" or "gefitinib" or "erlotinib" or "afatinib" or "osimertinib" or "dacomitinib" or "lapatinib") and ("retreatment" or "rechallenge" or "re-challenge" or "readministration")

(B) Cochrane
("NSCLC" or "Non-small cell lung cancer" or "non-small cell lung cancer") and ("TKI" or "tyrosine kinase inhibitors" or "anti-EGFR" or "EGFR" or "gefitinib" or "erlotinib" or "afatinib" or "osimertinib" or "dacomitinib" or "lapatinib") and ("retreatment" or "rechallenge" or "re-challenge" or "readministration")

(C) Embase
('nsclc' OR 'non-small cell lung cancer'/exp OR 'non-small cell lung cancer') AND ('tki' OR 'tyrosine kinase inhibitors' OR 'anti-egfr' OR 'egfr'/exp OR 'egfr' OR 'gefitinib'/exp OR 'gefitinib' OR 'erlotinib'/exp OR 'erlotinib' OR 'afatinib'/exp OR 'afatinib' OR 'osimertinib'/exp OR 'osimertinib' OR 'dacomitinib'/exp OR 'dacomitinib' OR 'lapatinib'/exp OR 'lapatinib') AND ('retreatment'/exp OR 'retreatment' OR 'rechallenge' OR 're-challenge' OR 'readministration')

(D) ESMO
(lung) and (tyrosine kinase inhibitor) and (retreatment OR rechallenge OR re-treatment OR re-exposure OR reexposure)

(E) ASCO
lung AND retreatment

ASCO: American Society of Clinical Oncology; ESMO: European Society for Medical Oncology. No filters were applied in any of the databases.

Table S3 List of studies excluded after full review

Author	Year	Title	Reason for exclusion
Nakashima [38]	2013	Clinical benefit of second EGFR-TKI retreatment on overall survival in patients with advanced non-small-cell lung cancer harboring EGFR-mutation positive after failure of the initial EGFR-TKI treatment: A retrospective analysis	No interval chemo and control group did not receive TKI after chemo
Takeda [39]	2015	Second-generation EGFR-TKI (afatinib) rechallenge to overcome acquired resistance to first-generation EGFR-TKIs	Lack of information regarding previous treatments before TKI
Kodani [40]	2013	Rechallenge with EGFR-TKI after a drug holiday, retrospective study in single institution	No interval chemo
Heon [41]	2012	Response to EGFR tyrosine kinase inhibitor (TKI) retreatment after a drug-free interval in EGFR-mutant advanced non-small cell lung cancer (NSCLC) with acquired resistance	Lack of information regarding previous treatments before TKI
Chi [42]	2022	EP08.02-134 Real-world Efficacy of Dacomitinib in Patients with Previously EGFR-TKI Treated Non-small Cell Lung Cancer	Lack of information regarding previous treatments before TKI
Yamaguchi [43]	2019	Re-challenge of afatinib after 1st generation EGFR-TKI failure in patients with previously treated non-small cell lung cancer harboring EGFR mutation.	Lack of data for the re-exposed population
Zhao [44]	2014	Mutation abundance affects the efficacy of EGFR tyrosine kinase inhibitor readministration in non-small-cell lung cancer with acquired resistance.	Lack of the outcomes of interest
Araki [45]	2024	EGFR-TKI rechallenge in patients with EGFR-mutated non-small-cell lung cancer who progressed after first-line osimertinib treatment: A multicenter retrospective observational study	Lack of data for the population receiving interval chemo
Yokouchi [46]	2007	Clinical benefit of readministration of gefitinib for initial gefitinib-responders with non-small cell lung cancer.	No interval chemo or lack of data regarding previous lines of therapy
Yamada [47]	2021	Effectiveness and Safety of EGFR-TKI Rechallenge Treatment in Elderly Patients with Advanced Non-Small-Cell Lung Cancer Harboring Drug-Sensitive EGFR Mutations.	Lack of data for the re-exposed population
Watanabe [48]	2011	Clinical responses to EGFR-tyrosine kinase inhibitor retreatment in non-small cell lung cancer patients who benefited from prior effective gefitinib therapy: a retrospective analysis.	TKI not as first-line
Tang [49]	2014	Different treatment orders achieved similar clinical results: a retrospective study for retreatment of epidermal growth factor receptor tyrosine kinase inhibitors in 120 patients with non-small-cell lung cancer.	No interval chemo or lack of data regarding previous lines of therapy
Tanaka [50]	2019	Real-world study of afatinib in first-line or re-challenge settings for patients with EGFR mutant non-small cell lung cancer.	No interval chemo or lack of data regarding previous lines of therapy
Tanaka [51]	2022	Clinical efficacy of dacomitinib in rechallenge setting for patients with epidermal growth factor receptor mutant non-small cell lung cancer: A multicenter retrospective analysis (TOPGAN2020-02).	Lack of data for the re-exposed population
Kwon [52]	2017	Predictive Factors for Switched EGFR-TKI Retreatment in Patients with EGFR-Mutant Non-Small Cell Lung Cancer.	Lack of data for the re-exposed population
Cho [53]	2015	Clinical efficacy of erlotinib, a salvage treatment for non-small cell lung cancer patients following gefitinib failure.	Lack of data for the re-exposed population
Han [54]	2017	A Phase II Study of Poziotinib in Patients with Epidermal Growth Factor Receptor (EGFR)-Mutant Lung Adenocarcinoma Who Have Acquired Resistance to EGFR-Tyrosine Kinase Inhibitors.	No interval chemo
Miyawaki [55]	2021	Rechallenge with previously administered epidermal growth factor receptor-tyrosine kinase inhibitors in EGFR-mutated non-small cell lung cancer with leptomeningeal metastasis.	No interval chemo
Vavalà [56]	2016	BE-POSITIVE: Beyond progression after tyrosine kinase inhibitor in EGFR-positive non small cell lung cancer patients: Results from a multicenter Italian observational study.	No interval chemo
Takahashi [57]	2019	P2.14-11 Retreatment with EGFR-TKI for 541 NSCLC Patients with EGFR Mutation	No interval chemo
Yoshino [58]	2013	Retrospective Analysis of Re-Challenge of EGFR TK-IS in Patients with Advanced and Recurrent Non-Small Cell Lung Cancer	No interval chemo

Sun [59]	2016	Gefitinib retreatment beyond progression in advanced NSCLC patients with sensitive EGFR mutations	No interval chemo
Sakamori [60]	2017	Comparison of afatinib versus erlotinib for advanced non-small-cell lung cancer patients with resistance to EGFR-TKI	No interval chemo
Ma [61]	2017	Recurrent response to advanced NSCLC with erlotinib developing central nervous system failure during gefitinib or icotinib treatment	No interval chemo
Acharya [62]	2021	P48.14 Metastatic NSCLC -Re-Challenging With Fist Generation TKI After a Drug Free Holiday After Resistance to 3rd Generation TKI	No interval chemo
Oda [63]	2017	Phase II Study of the EGFR-TKI Rechallenge With Afatinib in Patients With Advanced NSCLC Harboring Sensitive EGFR Mutation Without T790M: Okayama Lung Cancer Study Group Trial OLCSG 1403	No interval chemo
Kodama [64]	2021	Retrospective analysis of osimertinib re-challenge after osimertinib-induced interstitial lung disease in patients with EGFR-mutant non-small cell lung carcinoma	Lack of outcomes of interest
Kuiper [65]	2015	Rationale and study design of the IRENE-trial (NVALT-16): a phase II trial to evaluate iressa rechallenge in advanced NSCLC patients with an activating EGFR mutation who responded to an EGFR-TKI used as first-line or previous treatment	TKI not exclusively as first-line; no results available
Imaji [66]	2023	Safety and efficacy of osimertinib rechallenge or continuation after pneumonitis: A multicentre retrospective cohort study	Lack of outcomes of interest
Araki 67]	2023	Rechallenge of afatinib for EGFR-mutated non-small cell lung cancer previously treated with osimertinib: a multicenter phase II trial protocol (REAL study)	No results available
Marret [68]	2023	Treatment Patterns and Adverse Event-Related Hospitalization Among Patients with Epidermal Growth Factor Receptor (EGFR)-Mutated Metastatic Non-small Cell Lung Cancer After Treatment with EGFR Tyrosine Kinase Inhibitor and Platinum-Based Chemotherapy Regimens	Lack of outcomes of interest
Guo [69]	2022	The safety and efficacy of immune checkpoint inhibitor rechallenge after immune-related adverse events in stage IV NSCLC patients.	Lack of outcomes of interest

Table S4 Median OS (A) and median PFS (B) of individual studies for NSCLC patients treated with EGFR-rechallenge

(A) Median OS

Study	Median OS (95% CI) in months	N	TKI generation	TKI drug
Asahina 2010 [25]	14.7 (11.1–15.5)	15	First generation	Gefitinib
Cappuzzo 2016 [12]	10.2 (8.8–14.1)	58	First generation	Gefitinib
Chang 2017 [22]	12.6 (10.4–20.9)	205	First/second generation	Erlotinib/gefitinib/afatinib
Chen 2016 [27]	7 (NA)	71	First generation	NA
Y. Lee 2023 [29]	8.5 (NA)	63	First generation	Erlotinib/gefitinib
Kaira 2021 [21]	25 (NA)	62	First/second generation	Erlotinib/gefitinib/afatinib
Koizumi 2012 [28]	12 (8–16)	20	First generation	Gefitinib
Oh 2012 [32]	11.4 (7.8–13.7)	23	First generation	Gefitinib
Song 2019 [17]	10.3 (5.8–15.4)	46	First generation	Gefitinib
Song 2013 [33]	9.9 (7.5–12.2)	33	First generation	Erlotinib/gefitinib
Yu 2013 [37]	12 (NA)	38	First generation	Erlotinib/gefitinib
Oda 2018 [19]	11.6 (9.2–NA)	12	Second generation	Afatinib
Chen 2016 [27]	7.4 (NA)	9	Second generation	Afatinib
Ichihara 2019 [20]	9 (7.1-NA)	17	Third generation	Osimertinib
Soto Parra 2021 [35]	15 (NA)	54	Third generation	Osimertinib

OS: overall survival; CI: confidence interval; N: number of patients; TKI: tyrosine kinase inhibitors; NA: not available/applicable.

(B) Median PFS

Study	Median PFS (95% CI) in months	N	TKI generation	TKI drug
Asahina 2010 [25]	2.5 (1.6–3.2)	15	First generation	Gefitinib
Becker 2011 [26]	6.5 (1–16+)	14	First generation	Erlotinib
Cappuzzo 2016 [12]	2.8 (2.4–3.1)	58	First generation	Gefitinib
Chang 2017 [22]	4.1 (2.7–4.6)	205	First/second generation	Erlotinib/gefitinib/afatinib
Y. Lee 2023 [29]	2.8 (NA)	63	First generation	Erlotinib/gefitinib
Kaira 2021 (A) [21]	5 (NA)	62	First/second generation	Erlotinib/gefitinib/afatinib
Kaira 2021 (B) [21]	2 (1–9.5)	13	First/second generation	Erlotinib/gefitinib/afatinib
Koizumi 2012 [28]	2 (0.9-3.1)	20	First generation	Gefitinib
Song 2019 [17]	4.4 (3.2–4.8)	46	First generation	Gefitinib
Song 2013 [33]	1.5 (0.6–2.3)	33	First generation	Erlotinib/gefitinib
Yu 2012 [37]	3 (NA)	38	First generation	Erlotinib/gefitinib
Xia 2014 [36]	6 (NA)	27	First generation	Erlotinib/gefitinib
Oda 2018 [19]	4.2 (2–5.8)	12	Second generation	Afatinib
Soto Parra 2021 [35]	9 (NA)	54	Third generation	Osimertinib
Ichihara 2019 [20]	4.1 (1.9–6.7)	17	Third generation	Osimertinib

PFS: progression-free survival; CI: confidence interval; N: number of patients; TKI: tyrosine kinase inhibitors; NA: not available/applicable; Kaira (A): refers to the group of patients treated with PD-1 blockade before TKI rechallenge; Kaira (B) refers to the group of patients who did not receive PD-1 blockade before rechallenging.

Table S5 Median duration of response of TKI rechallenge according to individual studies

Study	Median duration of response in months
Cappuzzo 2016 [12]	3.6 (95% CI: 3–5.6)
Y. Lee 2023 [29]	2.2 (NA)
Uy 2023 [31]	3.6 (range: 1–10.3)
Oda 2018 [19]	4.4 (range: 1.2–18)
Tomizawa 2009 [34]	4 (range: 0.9–19.8)

Data is given according to the information available in studies in range or 95% CI as indicated above.
TKI: tyrosine kinase inhibitors; CI: confidence interval; NA: not available.

Table S6 Efficacy outcomes according to the TKI-free interval between the first-line and rechallenge TKI for individual studies

Study	N	Outcome analyzed	Data cutoff	Reported result
Chang 2017 [22]	205	ORR, DCR, PFS	A 7 mos cut-off level was used	ORR:<br><7 mos: 3.6% (4/110 patients)<br>≥7 mos: 11.6% (11/95 patients)<br>p=0.034<br>DCR:<br><7 mos: 33.6% (37/110 patients)<br>≥7 mos: 56.8% (54/95 patients)<br>p=0.001<br>Multivariable analyses for PFS:<br>interval < 7mos was considered reference<br>HR: 0.61 (0.43–0.85), p<0.01
Koizumi 2012 [28]	20	Response rate	NA	No correlation between response to the second gefitinib and the length of interval of gefitinib treatment was found (6.8 ± 4.3 months in mean (standard deviation) vs. 6.2 ± 2.7 mos)
Lee 2014 [30]	68	Survival	NA	Multivariate analysis found that the interval from first EGFR-TKI failure to second EGFR-TKI start was an independent predictor for death after second EGFR-TKIs treatment<br>HR: 1.05 (95% CI 1.02-1.08), p<.001
Oda 2018 [19]	12	PFS	Median EGFR-TKI-free period of 9.6 months was used	> 9.6 mos (N=6): median PFS of 5.4 mos<br>< 9.6 mos (N=6): median PFS of 3.6 mos<br>p=0.1316
Tomizawa 2009 [34]	20	Response rate	Median EGFR-TKI-free period of 217 days was used	DCR:<br><217 days: 6 patients achieved DCR<br>>217 days: 7 patients achieved DCR<br><br>There was no correlation between responses to the second gefitinib and the length of the interval of gefitinib treatment.

TKI: tyrosine kinase inhibitors; N: number of patients; ORR: objective response rate; DCR: disease control ratte; PFS: progression-free survival; mos: months; CI: confidence interval; HR: hazard ratio; NA: not available; > greater than; < less than.

Table S7 Quality assessment of studies using the risk of bias summary for non-randomized studies (ROBINS-I) tool

Study	Bias due to confounding	Bias in selection of participants	Bias in classification of interventions	Bias due to deviations from intended interventions	Bias due to missing data	Bias in measurement of outcomes	Bias in selection of the reported result	Overall risk of bias judgment
Retrospective cohorts								
Becker 2011 [26]	Serious[a]	Low	Low	NA[b]	Low	Low	NA[b]	Serious
Chang 2017 [22]	Moderate	Low	Low	NA[b]	Low	Low	NA[b]	Moderate
Chen 2016 [27]	Moderate	Low	Low	NA[b]	Low	Low	NA[b]	Moderate
Ichihara 2019 [20]	Moderate	Low	Low	NA[b]	Low	Low	NA[b]	Moderate
Kaira 2021 [21]	Serious[a]	Low	Low	NA[b]	Low	Low	NA[b]	Serious
Lee 2014 [30]	Moderate	Low	Low	NA[b]	Low	Low	NA[b]	Moderate
Uy 2023* [31]	Serious[a]	Low	Low	NA[b]	Low	Low	NA[b]	Serious
Song 2013 [33]	Moderate	Low	Low	NA[b]	Low	Low	NA[b]	Moderate
Tomizawa 2009 [34]	Serious	Low	Low	NA[b]	Low	Low	NA[b]	Serious
Yu 2013 [37]	Moderate	Low	Low	NA[b]	Low	Low	NA[b]	Moderate
Prospective clinical trials								
Asahina 2010 [25]	Moderate	Low	Low	Low	Low	Low	Low	Moderate
Cappuzzo 2016 [12]	Moderate	Low	Low	Low	Low	Low	Low	Moderate
Y. Lee 2023* [29]	Moderate	Low	Low	NA[b]	Low	Low	NA[b]	Moderate
Koizumi 2012 [28]	Moderate	Low	Low	Low	Low	Low	Low	Moderate
Oda 2018 [19]	Moderate	Low	Low	Low	Low	Low	Low	Moderate
Oh 2012 [32]	Moderate	Low	Low	Low	Low	Low	Low	Moderate
Song 2019 [17]	Moderate	Low	Low	Low	Low	Low	Low	Moderate
Soto Parra 2021* [35]	Moderate	Low	Low	Moderate[c]	Low	Low	NA[b]	Moderate
Xia 2014 [36]	Moderate	Low	Low	Low	Low	Low	Low	Moderate

NA: no data available; *Abstract or conference presentations; [a] Non-randomized studies are likely biased due to confounding factors. Retrospective cohorts lacking adjustments for confounders were judged to be at serious risk of bias. Retrospective studies were judged at moderate risk of bias if important confounding factors (e.g., time between initial TKI and rechallenge, number of chemo cycles between initial and rechallenge) were identified and controlled for. Prospective studies are less likely to have sources of bias and confounding than retrospective studies. Therefore, they were classified as moderate risk of bias, even if no adjusted for confounding analysis was performed; [b] These retrospective studies or abstracts from conference presentations lacked a detailed description of the methods section. Hence, we could only partially assess the deviation from intended interventions and the selection of reported results; [c] This study was interrupted early due to a low enrolment rate.

Figures

Figure S1 DCR during rechallenge in the subgroup of patients who previously achieved DCR with initial TKI

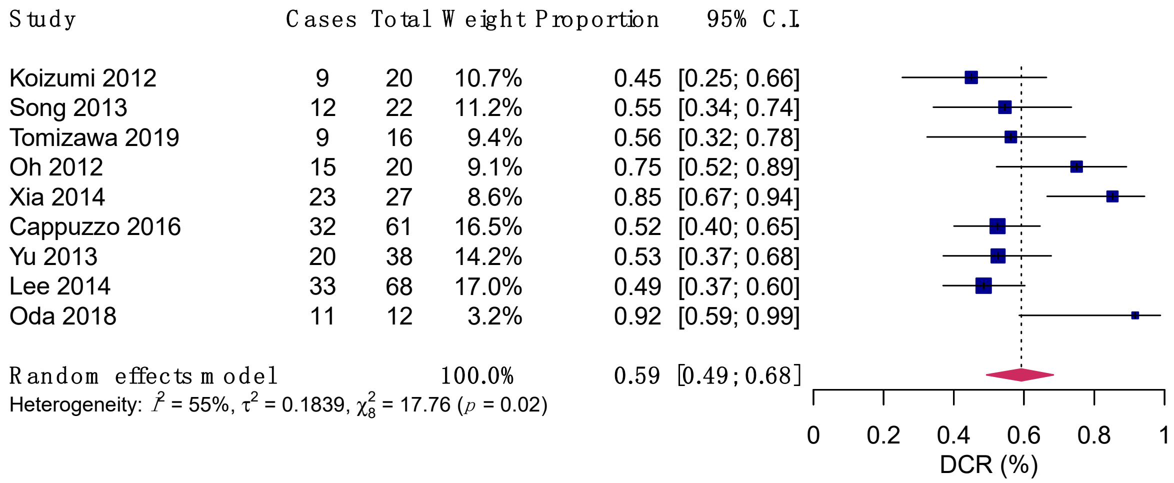

Proportions for each study are represented by a square and the horizontal line crossing the squares indicates the 95% confidence interval. The diamonds represent the estimated overall effect of the meta-analysis using random effects model [18,19,28,30,32–34,36,37].

Figure S2 Frequency of adverse events stratified by grade: (A) skin toxicity; and (B) diarrhea

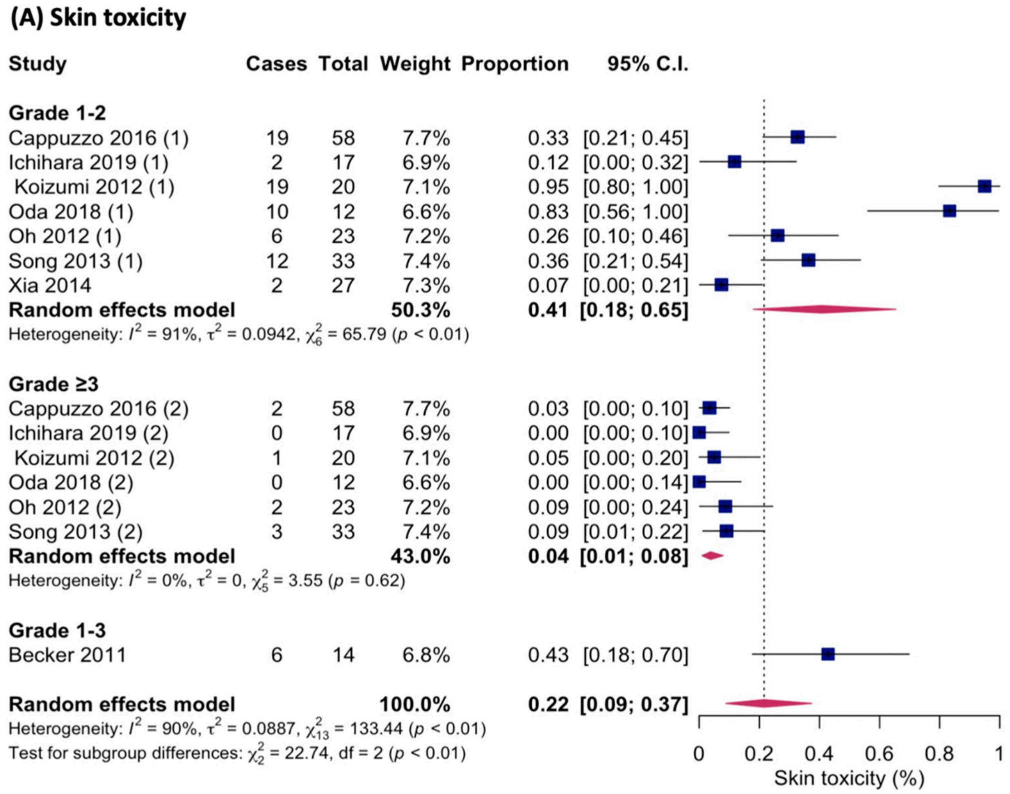

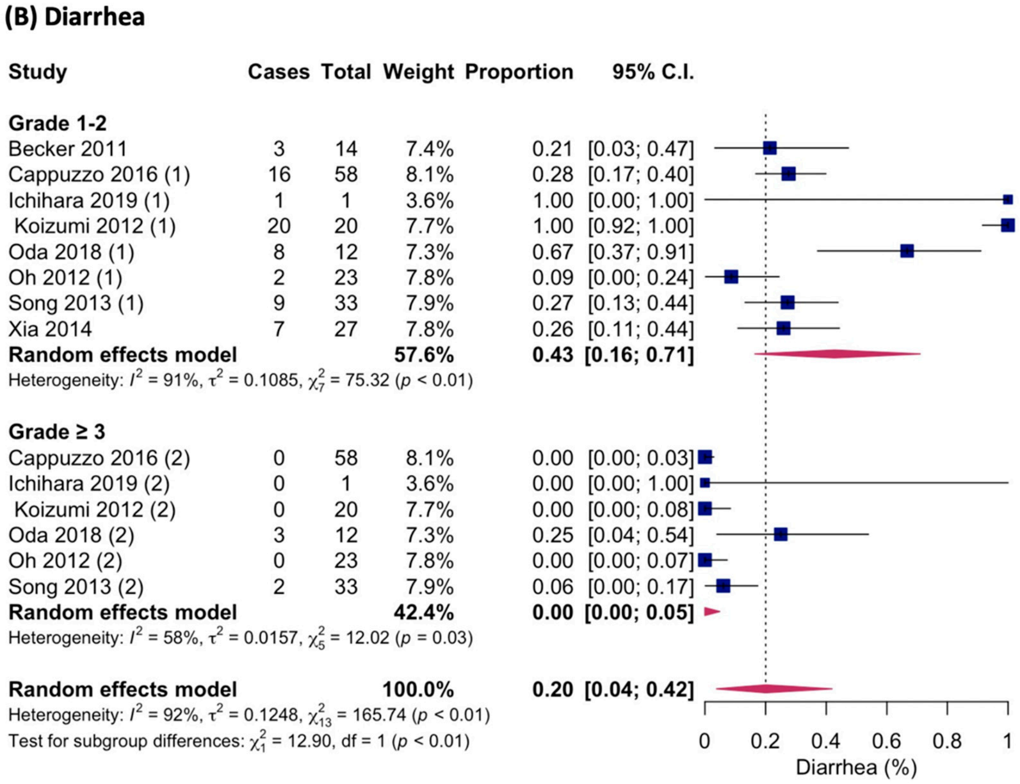

Proportions for each study are represented by a square and the horizontal line crossing the squares indicates the 95% confidence interval. The diamonds represent the estimated overall effect of the meta-analysis using random effects model; (1) Indicates analyses for grade 1-2 events and (2) indicates analyses for grade 3 or higher [18–20,25,26,28,32,33,36].

Figure S3 Funnel plot analysis of objective response rate (ORR) in NSCLC patients rechallenged with TKI

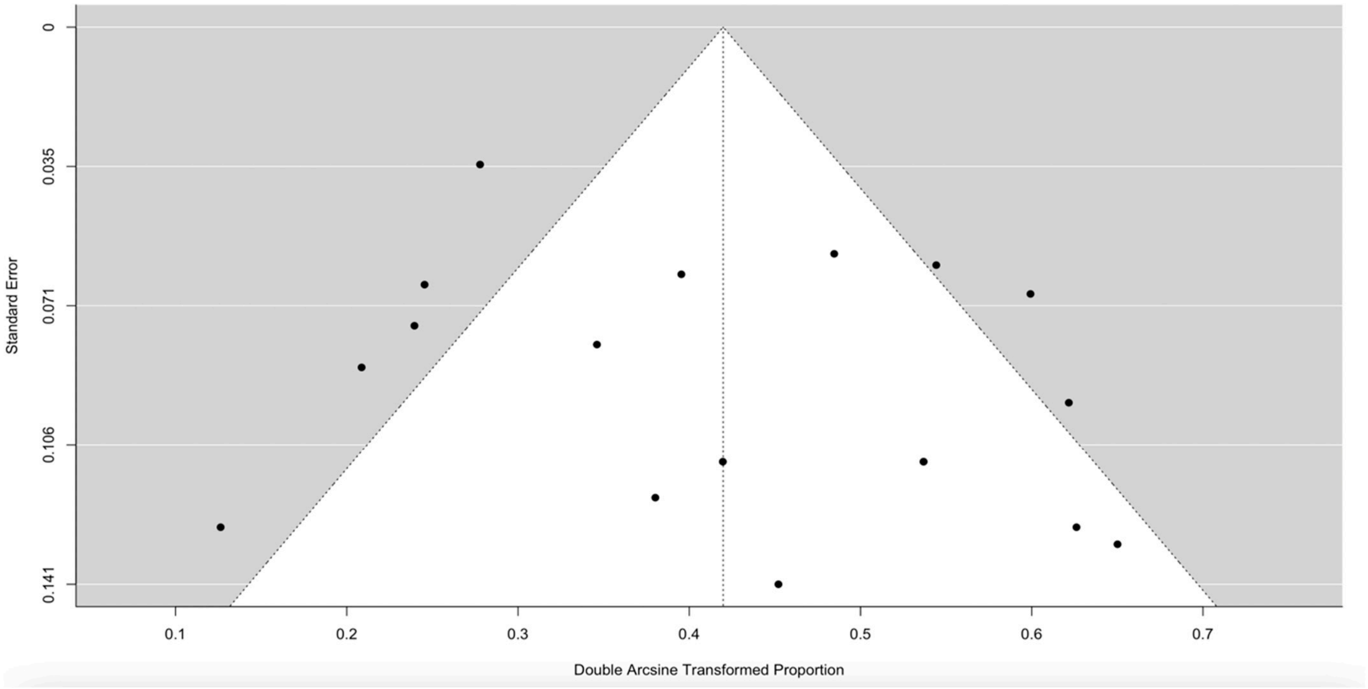

The dots represent individual studies, the effect size is represented in the x-axis and their corresponding error is in the y-axis. The central line represents the summary effect estimate [17–22,25,26,28–37].
